# Novel Epidemiologic Features of High Pathogenicity Avian Influenza Virus A H5N1 2.3.3.4b Panzootic: A Review

**DOI:** 10.1155/2024/5322378

**Published:** 2024-09-27

**Authors:** Carlos Sacristán, Ana Carolina Ewbank, Pablo Ibáñez Porras, Elisa Pérez-Ramírez, Ana de la Torre, Víctor Briones, Irene Iglesias

**Affiliations:** ^1^ Centro de Investigación en Sanidad Animal (CISA-INIA) Spanish National Research Council (CSIC), Madrid, Valdeolmos, Spain; ^2^ VISAVET Health Surveillance Centre Faculty of Veterinary Medicine Complutense University of Madrid, Madrid, Spain

**Keywords:** birds, conservation, emerging disease, epidemiology, influenza, mammals, wildlife, zoonosis

## Abstract

Avian influenza is one of the most devastating avian diseases. The current high pathogenicity avian influenza (HPAI) A virus H5N1 clade 2.3.4.4b epizootic began in the 2020–2021 season, and has caused a panzootic, considered one of the worst ever reported. The present panzootic has novel epidemiological features that represent a challenge for its prevention and control. This review examines key epidemiological changes of the disease such as seasonality, geographic spread, and host range. The seasonality of the virus has changed, and contrary to previous avian influenza epizootics, this subclade was able to persist during boreal summer. Its geographic range has expanded, with reports in all continents except Australia. During this epizootic, HPAIV H5N1 has broadened its host range, infecting hundreds of bird species, and causing the death of thousands of wild birds and over 300 million poultry. The number and diversity of mammal species infected by H5N1 2.3.4.4b is unprecedented. Although considered low, this strain's potential to spillover to humans should not be underestimated, especially considering the current extremely high viral circulation in animals and increasing adaptation to mammals. Overall, HPAI A(H5N1) clade 2.3.4.4b represents an ongoing and growing threat to poultry, wildlife, and human health.

## 1. Introduction

Influenza A viruses are enveloped, negative-sense, single-stranded, and segmented RNA viruses of the genus *Alphainfluenzavirus*, family *Orthomyxoviridae* [1, 2]. These viruses are classified according to their hemagglutinin (HA) and neuraminidase (NA) surface proteins into 18 and 11 subtypes, respectively [2]. Their genome organization into eight segments favors the reassortment of different influenza viruses coinfecting the same host cell, while their error-prone replication promotes the occurrence of mutations—characteristics that promote high evolutionary speed, and the emergence of novel viral subtypes and clades [1–3]. In birds, 16 HA and 9 NA types of avian influenza type A viruses (AIVs) have been reported, with 144 possible subtype combinations (e.g., H5N8); some of them are able to cause a viral disease named avian influenza or “bird flu” [4].

Avian influenza viruses are classified into low pathogenicity (LPAI) and high pathogenicity (HPAI) viral strains, according to their ability to cause severe disease and mortality in chickens in laboratory settings, and the detection of multibasic cleavage sites (MBCS) in the HA protein [1, 5]. LPAI viruses (LPAIVs) of subtypes H5 and H7 can naturally transform into HPAI viruses (HPAIVs) through the spontaneous acquisition of nucleotides coding for basic amino acids at the HA cleavage site [6–8]. This allows the processing of HA by ubiquitously expressed proteases enabling the systemic spread of the virus in the host [6, 7]. While most of the AIVs subtypes are LPAIV and cause no signs or mild disease, HPAIV (i.e., certain H5 and H7 subtypes) can cause severe disease and high mortality in infected wild birds and poultry; thus comprised into the former list A of diseases of the World Organisation for Animal Health (WOAH), which included transmissible diseases that can spread rapidly, have significant socioeconomic or public health impacts, and are critically important in the international trade of animals and animal products, and whose presence in a territory should be declared to that organization [1, 9]. Historically, HPAIVs were reported in poultry, not in wild birds [10]. Previous to 2002, the only exception was a common tern (*Sterna hirundo*) mass mortality event recorded in the Cape province, in South Africa in 1961, attributed to virus A/tern/South Africa/1961 (H5N3) [11–13]. This situation changed with the emergence of the H5N1 subtype, which spilled over from domestic geese to wild birds. Since then, this subtype has caused high morbidity and mortality rates both in wild and domestic birds [10, 14].

The H5N1 subtype is one of the most concerning HPAIVs worldwide [15], spread by poultry and migratory bird movements [16]. Since 2020, this virus—specifically HPAIV A (H5N1) clade 2.3.4.4b has changed its incidence and seasonal occurrence pattern, persisting during spring and summer months in the northern hemisphere [17–19]. The changes in seasonality have facilitated the spread of this viral clade through wild birds' migratory routes that previously did not significantly contribute to its dissemination [20]. Therefore, the current HPAIV A (H5N1) clade 2.3.4.4b has expanded its geographic and host range, spreading to countries where the virus had never been reported before, infecting not only avian species, but also mammals [10, 17, 20–23]. Thus, the HPAIV A (H5N1) clade 2.3.4.4b represents an ongoing and increasing threat to poultry, wildlife, and human health worldwide, as well as a considerable economic problem for the global poultry industry [24–27].

Herein, we briefly review and discuss the most remarkable novel epidemiological features—particularly temporal and geographical trends of the emerging HPAIV A (H5N1) clade 2.3.3.4b, responsible for most of the cases of the ongoing HPAI panzootic. Additionally, we analyze the number of HPAIV A (H5N1) notifications in animals according to class (aves or mammalia) and family, as recorded by the World Animal Health Information System [28] of the World Organization for Animal Health (WAHIS-WOAH).

## 2. Persistence in Summer Months

The occurrence of both LPAIVs and HPAIVs has traditionally shown a seasonal pattern, with a higher number of detections recorded between November and May (boreal winter and spring) in Europe [29], Asia [30], and North America [10]. Until 2021, HPAIV infections typically decreased in spring [17], likely due to the reduced environmental persistence of the virus under higher temperatures [31]. Additionally, Xu et al. [32] suggested that the waterfowl boreal autumn migration played a more significant role in the AIV transmission than spring migration, since (i) in autumn, there are summer-born individuals, which are immunologically naïve and thus particularly susceptible to AIV infection; (ii) the migratory waterfowl density is higher in autumn due to summer-born birds; and (iii) the autumn migration is more flexible and less synchronized compared to the spring migration.

An interesting shift was observed during 2021, 2022, and 2023 seasons, when HPAIV A (H5N1) 2.3.4.4b showed an unusual and remarkable persistence in wild birds over the summer, especially during 2022 [10, 28, 33, 34]. This indicates its potential for further outbreaks in the upcoming months, for instance, when naïve aquatic birds born in that same year arrive at their wintering areas in Europe, following their autumn migration [17, 18, 35]. In these summer seasons, the virus was most commonly detected in seabird breeding colonies of northwestern Europe [17, 35]. For instance, two successive mass mortality events caused by HPAIV A (H5N1) 2.3.4.4b were reported in great skuas (*Stercorarius skua*) in Scotland, spanning from April to November 2021, and May to August 2022 [26, 36].This unusual trend, with numerous outbreaks during boreal spring and summer, was also of concern in Africa ([1072021,33872022] and 1965 outbreaks [2023]) and Asia: ([48232021,6032022] and 5262 outbreaks [2023]) [28]. Additionally, in the Americas, H5N1 cases were also documented during boreal spring and summer of 2022 and 2023, with 661 and 1968 cases in wild birds, respectively [28]. Hicks et al. [31] found that, although geographic AIV dispersal between regions was strongly limited by physical distance, higher rates were associated with higher summer temperatures. Thus, the authors suggested that regions with warmer summers were more likely to act as sources of the virus to other regions, because summer temperatures may be a proxy for other environmental, temporal, or behavioral characteristics (e.g., timing of breeding and migration).

The persistence of HPAIV A (H5N1) observed in summer months represents a major seasonal variation that could have been one of the main drivers in the spread of the disease across different geographic ranges, along with the increased incidence, as suggested by Hicks et al. [31].

## 3. Geographic Range

### 3.1. Background Information

**Figure 1:**
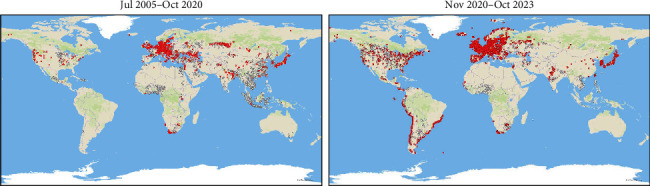
Notifications of HPAI in domestic birds (white dots), captive wild birds (yellow dots), and free-ranging wild birds (red dots) worldwide, from July 2005 to October 2020 (A), and from November 2020 to October 2023 (B). Source: DashFLUboard (CISA-INIA, CSIC). Available at: https://sgaicsic.maps.arcgis.com/apps/dashboards/8fd044ccc072431e919133b2949f350f (accessed on: 9/02/2024).

The H5N1 subtype first emerged in domestic waterfowl (geese) in southern China in 1996, and was named A/goose/Guangdong/1/1996 (H5N1) [37]. In 1997, the virus caused poultry outbreaks in China and Hong Kong, with associated human cases and mortality in the latter [38]. After several years undetected, H5N1 reemerged in 2003, causing poultry outbreaks across Asia [38]. Soon after, in 2005, the virus established a reservoir in aquatic wild birds [39]. These species contributed to the unprecedented spread of the virus to poultry from Asia to Europe, Africa, and the Middle East in 2005–2006, while previous HPAIV dissemination episodes were always linked to poultry [15, 38, 40]. Among HPAI A (H5) viruses, one of the most important clades and the most prevalent worldwide is H5 2.3.4.4, identified for the first time in China, in 2004 [41]. The clade 2.3.4.4 evolved into eight clades (a-h) [42]. The subtype H5N8 2.3.4.4 was first described in China, in 2010 [43], and emerged in the winter of 2013/2014 in South Korea, rapidly spreading to Japan, North America, and Europe between autumn 2014 and spring 2015 [41]. The first documented entry of H5 in the Americas was in 2014; in this epizootic, the subtype H5N8 clade 2.3.4.4b was introduced by migratory birds from Asia to the Pacific America flyway, and subsequently, to the Central and Mississippi flyways, affecting both wild and domestic birds [44, 45] (Figure 1).

**Figure 2:**
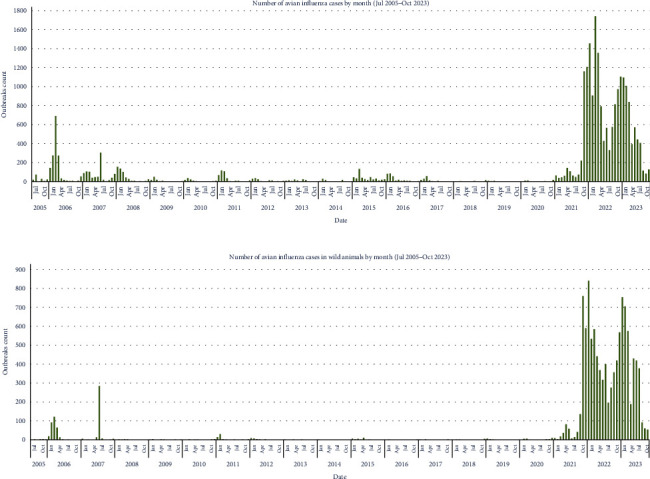
Number of HPAIV A (H5N1) cases in poultry and wildlife—including wild birds and wild mammals (A) and in wildlife (B) by month, from July 2005 to October 2023), based on the analysis of the World Organisation for Animal Health-World Animal Health Information System (WOAH-WAHIS, https://wahis.woah.org/#/home) database.

The dimension of the current H5N1 panzootic can be visualized in Figure 2, that shows the number of recorded notifications in poultry and wildlife (a) and in wildlife (b) of HPAIV A (H5N1) by month, from July 2005 to August 2023.

### 3.2. Current H5N1 2.3.4.4b Panzootic

In October 2020, H5 HPAIV infections were detected in Eurasian wigeons (*Mareca Penelope*) in the Netherlands; when sequenced, one of the complete genomes belongs to H5N8 and three as H5N1, all of the clade 2.3.4.4b [46]. This was the first report of H5N1 subclade 2.3.4.4b, and the strain seems to be the result of the reassortment between H5 HPAIVs and local European LPAIVs [46, 47]. In late spring 2021, H5N1 virus belonging to clade 2.3.4.4b and presenting a wild bird-adapted N1 NA gene emerged in Europe and became dominant, while a decrease in the H5N8 cases was recorded [38, 47] (for a more comprehensive description of the European situation, please refer to Fusaro et al. [48]). By the end of that year, this virus—that already presented several genotypes became the dominant circulating virus in Asia, Africa, Europe, and the Middle East [38, 49, 50], and was first detected in birds in North America: (i) in Canada, in poultry in December 2021, and retrospectively, in free-living great black-backed gull (*Larus marinus*) in late November 2021; (ii) in the United States of America (USA), in January 2022, in wild waterfowl; and (iii) in Mexico, in October 2022, in a captive Gyr falcon (*Falco rusticolus;*Table 1) [51–53]. Most of the HPAIV A (H5N1) 2.3.4.4b lineages identified in North America originated in Europe; however, the H5N1 2.3.4.4b that infected a bald eagle (*Haliaeetus leucocephalus*) in eastern Canada, in February 2022, was related to the virus isolated in Hokkaido, Japan, in January of that same year [54]. The continuous HPAIV A (H5N1) 2.3.4.4b outbreaks reported in wild and domestic birds in USA and Canada from May to September 2022 [28, 55], likely played a crucial role in triggering the unprecedented presence of the disease in South America. During the southern migration of birds in September, the virus spread further, leading to the first detections in Colombia in October, followed by Peru in November, and Central America (specifically Panama) in December 2022 [28, 56]. By February 2024, the virus had been reported in Central America (Costa Rica, Panama, Guatemala, Honduras), South America (Argentina, Bolivia, Brazil, Chile, Colombia, Ecuador, Falkland/Malvinas Islands, Paraguay, Peru, Uruguay, and Venezuela) and the Caribbean (Cuba; Table 1). This is an unprecedented situation, since HPAIVs had never been reported in wild birds in Mexico or in wild birds and poultry in Central America, South America, or the Caribbean [57], with the exception of H7N3 HPAIV that affected poultry in Chile in 2002 [58]. In October 2023, H5N1 2.3.4.4b was reported in brown skuas (*Stercorarius antarcticus*) and kelp gulls (*Larus dominicanus*) of South Georgia, in the Antarctic [59] (ProMED Mail Archive N° 20231024.8712796). To this date, the only geographic areas free of HPAIV A (H5N1) clade 2.3.4.4b in the world are Oceania and some oceanic islands.

### 3.3. Host Range

Avian influenza virus susceptibility and dispersal have been associated with a variety of factors such as bird host characteristics (e.g., host community species composition and host phylogenetic relatedness) [60], ecological features (foraging methods, migratory behaviors and distance, breeding distribution) [61], and environmental variables (e.g., land use, temperature, fecal pH, altitude, distance to water, habitat water salinity, and precipitation) [61–66]. Subsequently, we will review the main epidemiological characteristics of H5N1 in birds and mammals, with emphasis in wildlife.

### 3.4. Birds

**Figure 3:**
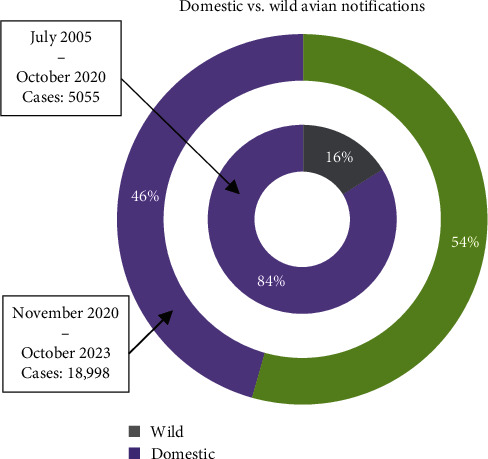
Percentage of HPAIV A (H5N1) notifications in domestic and wild birds between July 2005 and October 2020, and between November 2020 and October 2023, based on the World Organisation for Animal Health-World Animal Health Information System (WOAH-WAHIS, https://wahis.woah.org/#/home) database.

From July 2005 to October 2020, most of the HPAIV notifications came from domestic birds [28] (Figure 3). Nevertheless, between November 2020 and October 2023, 54% of the notifications corresponded to wild birds (Figure 3).

**Figure 4:**
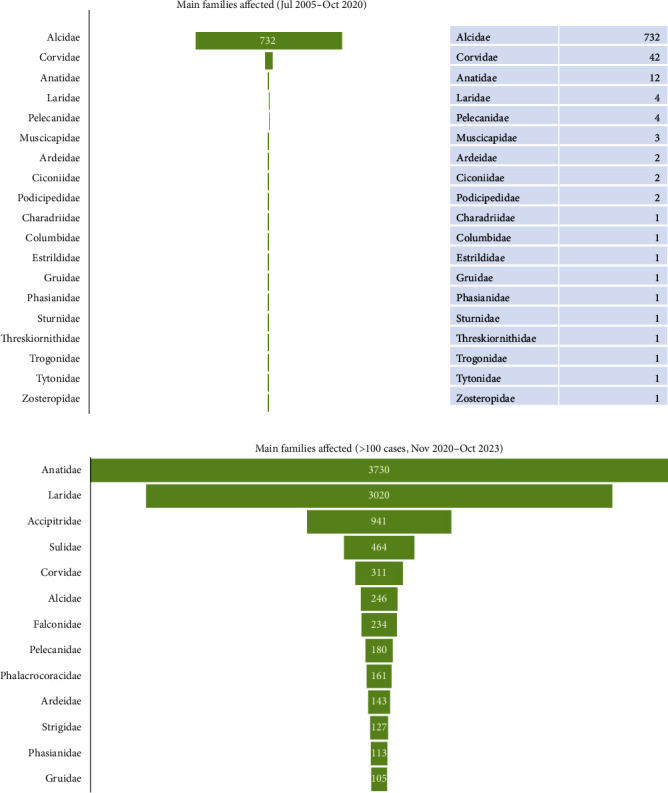
Families of birds with HPAIV A (H5N1) outbreaks in the World Organisation for Animal Health-World Animal Health Information System (WOAH-WAHIS, https://wahis.woah.org/#/home) database in the periods (A) July 2005–October 2020; and (B) between November 2020–October 2023 (only those families with over 100 registered outbreaks).

During the period comprised between July 2005 and October 2020, the virus was confirmed in wild birds of 33 species from 19 avian families (Figure 4A) of 11 orders.

According to Hill et al. [67], the HPAIV A (H5) subtype affects a broad range of avian hosts, particularly Anseriformes and Charadriiformes, but also Galliformes and nonaquatic bird species (e.g., passerines and raptors). After that, according to WOAH-WAHIS [28], since November 1, 2020 to November 1, 2023 (the beginning of the next influenza season), H5N1 cases were reported in 383 avian species, comprising 52 families and 25 orders. This represents that, since the emergence of 2020 H5N1 panzootic, the virus has been detected in ~3.5% (383/11001) of bird species, 20.6% (52/253) of avian families, and 56.8% (25/44) of the total number of bird orders, according to the world bird list of the International Ornithological Committee [68].

Since November 1, 2020, 13 wild avian families were the most affected (each one with more than 100 notifications), in descending order: Anatidae, Laridae, Accipitridae, Sulidae, Corvidae, Alcidae, Falconidae, Pelecanidae, Phalacrocoracidae, Ardeidae, Strigidae, Phasianidae, and Gruidae [28] (Figure 4B). Among orders, the most affected were Pelecaniformes (fam. Pelecanidae, Sulidae, Phalacrocoracidae, and Ardeidae), followed by Charadriiformes (fam. Laridae and Alcidae), Anseriformes (fam. Anatidae), Passeriformes (fam. Corvidae), Accipitriformes (fam. Accipitridae), Falconiformes (fam. Falconidae), Strigiformes (fam. Strigidae), Galliformes (fam. Phasianidae), and Gruiformes (fam. Gruidae). The complete list of avian families infected by H5N1 (July 2005 to October 31, 2023) is available in Figure S1.

It is important to consider that AIV subtype distribution, spread, and spillover may be influenced by the host's taxonomic classification (i.e., genus and family) within these avian groups, the breeding distribution overlap (due to a larger population of immunologically naïve chicks and juvenile hosts during breeding months), and migratory behaviors (particularly in higher summer temperatures, likely associated with the avian life cycle [e.g., breeding]) [31, 67].

Of note, HPAIV A (H5N1) transmission is strongly associated with avian migration [10, 52], which facilitates viral dissemination across long distances and the consequent introduction of the virus into areas with naïve populations [66]. For instance, recent studies found that gulls (order Charadriiformes) play a key role in the interhemispheric spread of AIV [69, 70], and are capable of transporting HPAIV A (H5) more rapidly than any other avian host, possibly due to their long-distance pelagic movements and immuno-naïve status against this subtype [71, 72]. Although transcontinental spread of HPAIV A/goose/Guangdong/1/1996 H5 to Asia, Russia, Europe, Africa, and the Middle East, had been previously reported in 2005 and 2009 [40], there were no reports of H5Nx in the Americas until 2014/2015, when the subtype H5N8 clade 2.3.4.4b was first detected, following wild bird migration across the Bering Strait to the Pacific coasts of Canada and the USA through the Pacific flyway [73]. Most recently, in 2021, the HPAI A (H5N1) clade 2.3.4.4b. virus originated in northwestern Europe was detected on the Atlantic coast of Canada, implying that the virus had been carried across the Atlantic [52]. Although that was a previously known route for LPAI, this was the first report of such spread in HPAIVs [72, 74], possibly promoted by migratory wild birds using different migratory routes (i.e., Icelandic, Greenland/Arctic, pelagic routes directly across the Atlantic Ocean) [52]. Although flyway overlapping is an important epidemiological factor [75], once within a given migratory pathway, the exchange rate of AIV strains can be 4–13 times greater than between flyways [76].

The fast spread of H5N1 viruses of clade 2.3.4.4b to several previously unaffected areas of the globe and their successful summer persistence were likely facilitated by the constant evolution and by the reassortment of these viruses with local LPAIVs, which facilitated virus adaptation to wild bird species that were very rarely or never before affected by H5N1 (e.g., barnacle goose [*Branta leucopsis]*, sea birds), leading to massive die-offs [77].

The expansion of H5N1 clade 2.3.4.4b to new territories gave the virus the opportunity to infect novel naïve host species, e.g., California condors (*Gymnogyps californianus*) in the USA [78], peruvian pelicans (*Pelecanus thagus*), peruvian boobies (*Sula variegata*), and guanay cormorants (*Leucocarbo bougainvillii*) in Peru [79, 80], and brown skuas (*S. antarcticus*) in the Antarctic region (ProMED Mail Archive N° 20231024.8712796). The virus can cause large mortality outbreaks in wild birds and have a devastating impact in threatened species [79–82]. For instance, H5N1 clade 2.3.4.4b likely killed thousands of peruvian pelicans and peruvian boobies in Peru, both species classified as endangered in that country [79, 80]. Additionally, ~24,000 Cape cormorants (*Phalacrocorax capensis*) and over 300 African penguins (*Spheniscus demersus*) died in South Africa, likely due to HPAIV A (H5N1) [83]. The virus was also confirmed in three Cape cormorants found dead in a mass mortality event involving 6500 specimens in Namibia [82]. In Senegal, the virus was associated with the death of 750 great white pelicans (*Pelicanus onocrolatus*) [84]. This virus can have particularly devastating effects in breeding colonies, as observed in adult and chick sandwich terns (*Thalasseus sandvicensis*) in the Netherlands [85], and in sandwich terns, common terns (*S. hirundo*), and northern gannet (*Morus bassanus*) among other species—in the North Sea [77]. This is especially concerning in seabirds, which generally have low annual reproductive rates and long life spans [85]. Aside from direct mortality, the disease can also reduce breeding success, as observed in griffon vultures (*Gyps fulvus*) and bald eagles [86, 87].

The pathogenicity and virulence of HPAIVs in birds is out of the scope of this review. Nevertheless, it is necessary to mention that some wild bird species may sustain subclinical HPAIV infections, which may favor the silent spread of the virus [88, 89].

### 3.5. Mammals

In mammals, AIV infection and spread are facilitated by several virus characteristics, including receptor binding properties, pH of HA activation, and polymerase activity [90–94]. Additionally, viral adaptations that promote efficient replication and transmission have been recently suggested as factors involved in the increased occurrence of HPAIV A (H5) 2.3.4.4b clade in mammalian species: (i) HA mutations capable of changing binding properties (i.e., from the avian-type *α*2,3-linked sialic acid receptors to the mammalian-type *α*2,6-linked sialic acid receptor) [95, 96]; (ii) HA mutations that optimize viral stability in mammalian airways (i.e., fusion of the viral and endosomal membranes, and subsequent cytoplasmic release of the viral genome) [97]; and (iii) increased virus replication based on substitutions in the polymerase complex (e.g., E627K mutation in the polymerase basic protein 2, PB2) [98–100] and PB2-D701N adaptation [99, 101, 102].

The spillover of H5Nx viruses to mammals is not new. For instance, previous natural infections by H5N1 subtype were detected in Felidae (i.e., captive tigers [*Panthera tigris*], captive leopards [*Panthera pardus*] and cats [*Felis catus*]), in Viverridae (Owston's civet [*Chrotogale owstoni*] kept under human care), in Ochotonidae (plateau pika [*Ochotona curzoniae*]) and in Hominidae (in humans) [103–106]. Additionally, experimental infections were successful in Mustelidae (domestic ferrets [*Mustela putorius furo*], Muridae (mice), Suidae (domestic pigs [*Sus scrofa*], and Cercopithecidae (long-tailed macaques [*Macaca fascicularis*]) [105]. Moreover, infections by HPAIV A (H5N8) clade 2.3.4.4b have been observed in red foxes (*Vulpes vulpes*), and gray (*Halichoerus grypus*), and harbor seals (*Phoca vitulina*) [35, 100, 107, 108]. Recently, HPAIV A (H5N5) clade 2.3.4.4b was detected in red fox and raccoon (*Procyon lotor*) [35], and H5N6 clade 2.3.4.4 b was reported in dogs (*Canis lupus familiaris*) [109].

Despite previous descriptions of HPAIVs in mammals, the number of cases and diversity of species affected by H5N1 2.3.4.4b is unprecedented. One of the most interesting features of H5N1 2.3.4.4b is that it infects free-ranging wild species, similarly to HPAIV A (H5N8) clade 2.3.4.4b [100, 107, 108], and not only animals kept under human care [103–105]. This current HPAIV A (H5N1) wave has noticeably expanded its mammalian host and geographic ranges [102]. It has been detected in novel mammal species within the orders Carnivora (families Canidae, Felidae, Mephitidae, Mustelidae, Otariidae, Procyonidae, Phocidae, Ursidae), Didelphimorphia (fam. Didelphidae), Rodentia (fam. Muridae), and Cetartyodactyla (families Bovidae, Camelidae, Delphinidae, and Phocoenidae) in Europe, North America and/or South America [20, 35, 102, 110–114].

The confirmed infections by HPAIV A (H5N1) avian influenza in terrestrial mammals, aside from humans, comprises a species of the family Didelphidae (Virginia opossum [*Didelphis virginiana*]), a species of the family Sciuridae (Abert's squirrel [*Sciurus aberti*]), a species of the family Muridae (house mouse [*Mus musculus*], a species of the family camelidae (Alpaca [*Lama pa*cos], two species of the family Bovidae (goat [*Capra aegagrus hircus*] and cow [*Bos taurus*], and several species of terrestrial carnivores within the families Mustelidae (i.e., Eurasian badger [*Meles meles*], American mink [*Neovison vison*], Eurasian otter [*Lutra lutra*], North American river otter [*Lontra canadensis*], Southern river otter [*Lontra provocax*], European pine marten [*Martes martes*], Beech marten [*Martes foina*], European polecat [*M. putorius*], ferret), Ursidae (i.e., black bear [*Ursus americatus*], brown bear [*Ursus arctos*], Kodiak grizzly bear [*U. arctos horribilis*], Asian black bear [*Ursus thibetanus*], polar bear [*Ursus maritimus*]), Felidae (i.e., domestic cat, tiger, lion [*Panthera leo*], Amur leopard [*P. pardus orientalis*], Puma [*Puma concolor*], Caracal [*Caracal caracal*], Eurasian lynx [*Lynx lynx*], bobcat [*Lynx rufus*], fisher cat [*Pekania pennanti*]), Canidae (i.e., dog, red fox, arctic fox [*Vulpes lagopus*], bush dog [*Speothos venaticus*], common raccoon dog [*Nyctereutes procyonoides*], Japanese raccoon dog [*Nyctereutes viverrinus*], coyote [*Canis latrans*]), Procyonidae (i.e., raccoon, South American coati [*Nasua nasua*]), and Mephitidae (striped skunk [*Mephitis mephitis*]) [35, 110–115].

Of note, this virus has been also detected in marine mammals: in mustelids, cetaceans, and pinnipeds. In mustelids, it was detected in marine otters (*Lontra felina*) in Chile [23]. In cetaceans, HPAIV A (H5N1) was found in the families Delphinidae (common bottlenose dolphin [*Tursiops truncatus*] in the USA, common dolphin [*Delphinus delphis*], white-sided dolphin [*Lagenorhynchus acutus*] in Canada, Chilean dolphin [*Cephalorhynchus eutropia*] in Chile), and Phocoenidae (harbor porpoise [*Phocoena Phocoena*] in Sweden, Burmeister's porpoise [*Phocoena spinipinnis*] in Chile) 20, 21, 23, 116–119].

In pinnipeds, the first report of the high pathogenicity H5N1 clade 2.3.4.4b was in June–July 2022 [22], in Phocidae. Specifically, this clade was likely the cause of an unusual mortality event involving at least 164 harbor seals and 11 gray seals in Maine, New England, USA [22]. Most of the animals were found dead; however, some had presented respiratory and neurological signs [22]. Swab samples of some of these animals were tested by rtRT-PCR to avian influenza, and 48.6% (17/35) harbor and 33.3% (2/6) of gray seals tested positive to HPAIV A (H5N1) clade 2.3.4.4b [22]. The virus was also detected in southern elephant seals (*Mirounga leonina*, fam. Phocidae) in Argentina, likely involved in a mass mortality event recorded in that species [120], and was also found in southern elephant seals in South Georgia Island, and in an individual of the same species in the South Shetland Islands [121, 122].

In pinnipeds of the family Otariidae, a mass mortality event was described in Peru, in early February 2023 [21]. During that event, at least 630 South American sea lions (*Otaria flavescens*) and four South American fur seals (*Arctocephalus australis*) were found dying or dead. Nevertheless, the number of confirmed H5N1 cases was small. Only nine out of the 12 South American fur seals tested by rtPCR were positive to HPAIV A (H5N1) [21]. Additionally, another three South American sea lions and a pool of five individuals tested positive in the study conducted by Leguia et al. [20] in Peru. In Chile, 36 South American sea lions tested positive to H5N1 and died during mass mortality events, according to the national authorities [23, 123]. Subsequently, HPAIV A (H5N1) clade 2.3.4.4b cases have been described in South American sea lions and South American fur seals in the southwestern Atlantic, in Argentina, Uruguay, and Brazil [124–126] (ProMED Mail Archive N° 20231017.8712681). HPAIV A (H5N1) has also been confirmed in a northern fur seal (*Callorhinus ursinus*) in Russia [127] and in an Antarctic fur seal (*Arctocephalus gazella*) of South Georgia Island [121]. In spite of the high number of cases reported in marine mammals, the virus was likely acquired from birds, not through horizontal infection among mammals. Cases in terrestrial and marine mammals occur following wild bird epizootics [128]. The large number of cases described in wild mammals promotes adaptation of the virus to mammals. Nevertheless, mammal-to-mammal transmission was only reported in captive American minks [129].

#### 3.5.1. Natural Infections in Cats and Fur Farmed Animals

In domestic and fur farmed mammals, HPAIV (H5N1) clade 2.3.3.4b was reported in an outbreak in American minks in a fur farm located in northwestern Spain, in October 2022 [129]. Another one involving 20 fur farms housing American minks, raccoon dogs, and foxes (arctic and red fox, and their crossbreeds), was reported in Finland [130], and the number of cases continued to increase, with over 60 fur farms affected in that country [131]. In April 2023, five domestic dogs and one cat living on the premises of a backyard poultry farm with registered increased poultry mortality, in Italy, seroconverted the virus in the absence of clinical signs [113]. Two important outbreaks have affected domestic cats: one involving at least 25 cats from different regions of Poland [132], and another one affecting two cat shelters in South Korea [133]. In both cases the virus was highly pathogenic for the cats, causing acute respiratory and neurological signs that led to their death [132, 133]. Interestingly, in all outbreaks affecting cats and fur farmed animals, the virus presented one or more mammalian adaptive mutations in the PB2 gene—in proteins T271A [113, 129, 130], 526R [132], and 627K [130, 132], suggesting potential public health implications.

#### 3.5.2. Cases in Humans

According to the World Health Organization records, 887 human cases of influenza A (H5N1) infections were reported to the organization in 23 countries, including 461 deaths [134]. Of those, H5N1 2.3.4.4b infected at least seven people in the Americas, in Ecuador, the USA, and Chile [135, 136, 57], three in Asia (one in Vietnam and two in China), seven in Europe (two in Spain and five in the UK) and one imported case in Australia. Most of these cases occurred following close and prolonged contact with sick or dead poultry, except for a case in Chile, where the infection apparently resulted from environmental contamination due to the presence of a large number of dead wildlife on the beach where the patient lived [137], and at the US, with some cases following exposure to dairy cows [136]. Additionally, it is important to remark that some of the people that tested H5N1-PCR-positive likely presented H5N1 genetic material due to environmental contamination instead of sustaining an active infection [138].

## 4. Conclusions

The current HPAIV A (H5N1) 2.3.4.4b panzootic is unprecedented, showcasing a complex and multifaceted epizootiology, with interactions between wild birds, domestic poultry, mammals, the environment, and human activities. The epidemiological changes of the virus have revealed an unusual and remarkable disease dynamic, with significant implications for predicting and managing future outbreaks. The presence of different HPAIV A (H5N1) 2.3.4.4b genotypes resulting from mutations and the reassortment of these viruses with local LPAIVs should be closely monitored, considering that such high genetic diversity can enhance the virus' adaptation to novel host species.

Traditionally, avian influenza exhibited a marked seasonal pattern, with higher occurrences during the winter months in the northern hemisphere. Nevertheless, a notable shift occurred during the 2021, 2022, and 2023 seasons, with a noticeable number of outbreaks also reported during the summer months. Seasonal variations play a crucial role in influencing the spread of HPAIV A (H5N1) across different geographical ranges.

During the ongoing HPAIV A (H5N1) 2.3.4.4b panzootic, the virus rapidly spread across various regions, affecting numerous bird species. The current increased incidence of HPAIV A (H5N1) in wild birds implies a higher presence of virus in the environment, leading to associated risks:

First, the increased risk of virus introduction into poultry farms due to the critical interface between wild bird migration routes and areas of poultry production. This zone is particularly susceptible since HPAIV may entry through contact with infected wild birds, other infected domestic birds, or contaminated fomites. Thus, implementing robust biosecurity measures and monitoring the disease in wild birds is critical to early detection, response, and outbreak prevention in poultry farms. Second, the unprecedented cross-species infections. This virus' ability to infect a diverse range of avian and mammalian species, both in captivity and in the wild is unprecedented, and raises concerns about its potential impact on endangered species, thus increasing the risk of spillovers and posing a major conservation threat to vulnerable species. Third, the increased number of cases in domestic mammals. The current epizootic has demonstrated an increased risk of HPAIV infections in mammalian species, including fur farms in Spain (2022) and Finland (2023), as well as domestic cats in South Korea and Poland (2023). These outbreaks represent a major public health threat due to their close contact with humans. Fourth, the increased risk of human cases. Despite the historically low HPAIVs zoonotic risk, the recent intensification of epidemics, the high mutation rate of these viruses, and their frequent spillover to mammals increases its potential threat to public health. Thus, measures including education and awareness campaigns are important for minimizing the risk of zoonotic transmission to humans.

This study underscores the complexity and dynamic nature of avian influenza virus transmission, emphasizing the importance of understanding the interplay among virus dynamics, host ecology, and environmental factors. Effective and continuous surveillance, control strategies, and monitoring of the evolution and dynamics of avian influenza in domestic and wild birds, mammals, and humans are essential to mitigate the impact of HPAIV A (H5N1) 2.3.4.4b on both avian and mammal populations and to protect vulnerable species from the devastating consequences of this virus' spread. Continued research and intersectoral collaboration between international human and animal health organizations and wildlife authorities are essential to monitor the evolving epidemiology of H5N1 and to implement timely measures to safeguard the poultry industry, public health, and biodiversity.

## Figures and Tables

**Table 1:** First reports of HPAIV (A) H5N1 in the countries/territories of the Americas and in the Antarctic region during the current panzootic (November 1, 2020 to October 31, 2023).

Year	Month	Country
2021	December ^*∗*^	Canada

2022	January	United States of America
	October	Colombia, Mexico
	November	Peru
	December	Chile, Panama, Venezuela

2023	January	Costa Rica, Ecuador, Greenland, Honduras
	February	Argentina, Bolivia, Cuba, Guatemala, Uruguay
	May	Brazil, Paraguay
	October	Antarctic region (South Georgia Islands)

2023	October	Falkland/Malvinas Islands

^*∗*^H5N1 was retrospectively found in wild birds in Canada in late November, 2021 [52].

## Data Availability

All data are contained in the manuscript and in the supplementary file.

## Supporting Information

Supporting Information Figure S1: avian and mammalian families with notifications of HPAI (A)H5N1 outbreaks since July 2005 to October 2023, according to World Organisation for Animal Health-World Animal Health Information System (WOAH-WAHIS https://wahis.woah.org/#/home).
