# Correction to “Novel Epidemiologic Features of High Pathogenicity Avian Influenza Virus A H5N1 2.3.3.4b Panzootic: A Review”

**DOI:** 10.1155/tbed/9758482

**Published:** 2026-05-04

C. Sacristán, A. Ewbank, P. Ibáñez Porras, et al., “Novel Epidemiologic Features of High Pathogenicity Avian Influenza Virus A H5N1 2.3.3.4b Panzootic: A Review,” *Transboundary and Emerging Diseases*, 2024, no. 1 (2024): 13 pages, https://doi.org/10.1155/2024/5322378.

In the article, an error was identified in panel A of Figure 4, whereby “Indeterminatum” was incorrectly labelled as “Alcidae.” Additionally, the authors have updated the figure legend to clarify that only H5N1 cases notified to WOAH‐WAHIS were included in the figure. This error does not affect the results, interpretation, or conclusions of the study.

The correct panel A of Figure 4 and the updated Figure 4 legend is provided below:

**Figure 4:**
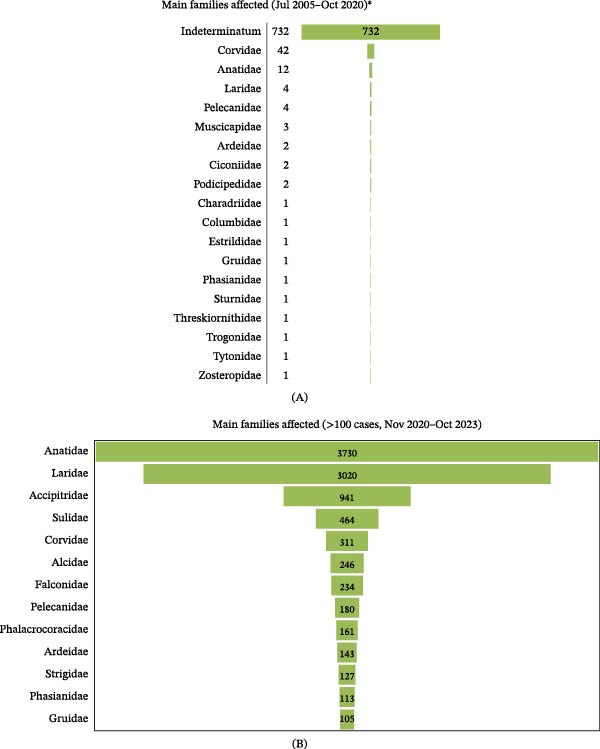
Families of birds involved in HPAIV A(H5N1) outbreaks according to the World Organisation for Animal Health ‐ World Animal Health Information System (WOAH‐WAHIS, https://wahis.woah.org/#/home) database in the periods (A) July 2005–October 2020; and (B) November 2020–October 2023 (only those families with over 100 registered outbreaks are listed).  ^∗^Please note that only confirmed HPAIV A (H5N1) cases notified to WOAH‐WAHIS were included in the figure. In the period July 2005–October 2020, a high number of notifications (especially for Anatidae) were limited to hemagglutinin characterization (e.g., H5), and for that reason, were not included in the figure.

[CORRECT Figure 4]

We apologize for this error.

