# Supplementary material for: Novel Epidemiologic Features of High Pathogenicity Avian Influenza Virus A H5N1 2.3.3.4b Panzootic: A Review
Source: Transbound Emerg Dis. 2024 Sep 27;2024:5322378. doi: 10.1155/2024/5322378 (PMC12016977; doi:10.1155/2024/5322378)
Supplement: Supporting Information — Figure S1: avian and mammalian families with notifications of HPAI (A)H5N1 outbreaks since July 2005 to October 2023, according to World Organisation for Animal Health-World Animal Health Information System (WOAH-WAHIS https://wahis.woah.org/#/home). [file 5322378.f1.docx]

**SUPPLEMENTARY FILES**


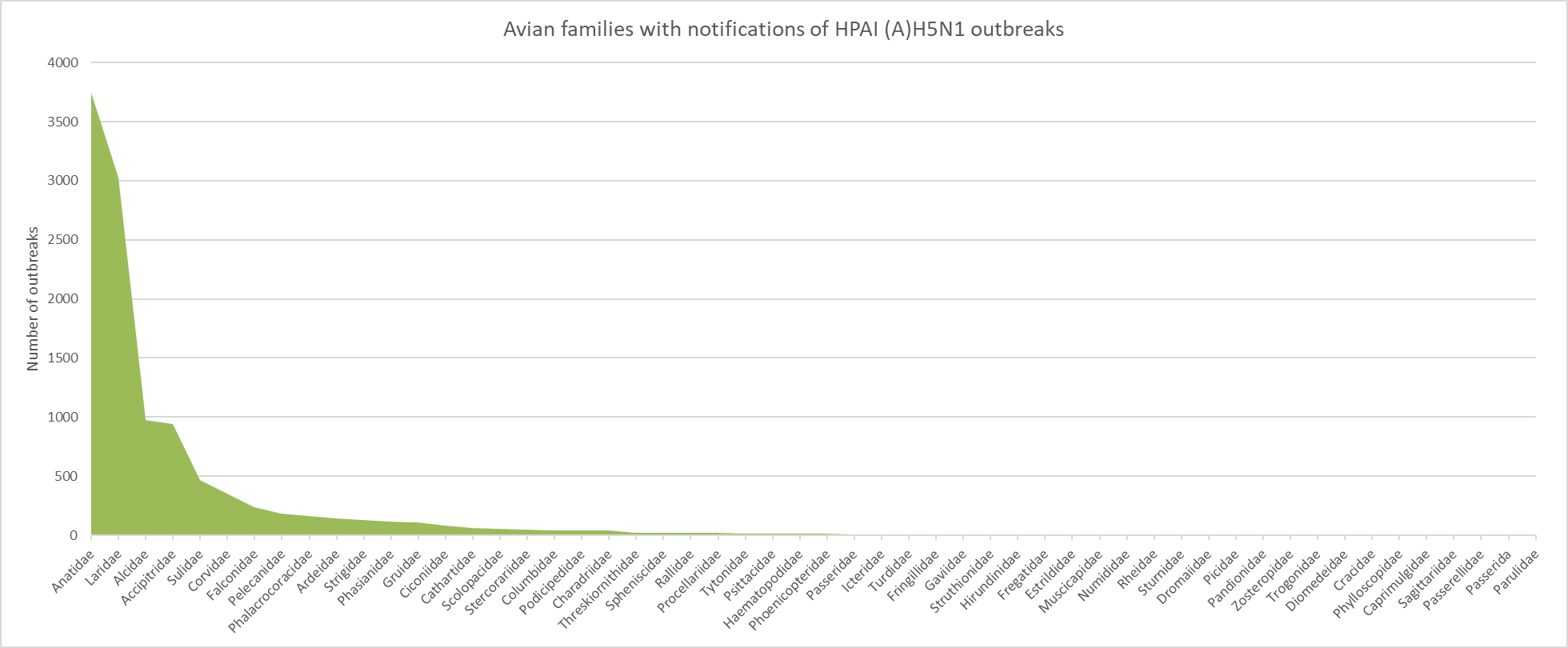


**Supplementary figure 1.** Avian and Mammalian families with notifications of HPAI (A)H5N1 outbreaks since July 2005 to October 2023, according to World Organisation for Animal Health-World Animal Health Information System (WOAH-WAHIS <https://wahis.woah.org/#/home>).
